# Effect of COVID-19 Italian Lockdown on Maxillofacial Trauma Related to Domestic Violence: A Retrospective Cohort Study

**DOI:** 10.3390/life12101463

**Published:** 2022-09-20

**Authors:** Francesco Ferragina, Ida Barca, Alfonso Sorrentino, Elvis Kallaverja, Sara Piloni, Antonella Arrotta, Maria Giulia Cristofaro

**Affiliations:** 1 Unit of Maxillofacial Surgery, Department of Experimental and Clinical Medicine, “Magna Graecia” University, Viale Europa, 88100 Catanzaro, Italy; 2 Unit of Maxillofacial Surgery, Department of Neurosciences, Reproductive and Odontostomatological Sciences, University Hospital of Naples “Federico II”, 80131 Naples, Italy; 3 “Magna Graecia” University, Viale Europa, 88100 Catanzaro, Italy

**Keywords:** epidemiology, maxillofacial surgery, maxillofacial trauma, domestic violence, interpersonal violence, COVID-19

## Abstract

Background: This retrospective study aims to identify the potential reasons for the increase in maxillofacial trauma from domestic violence in the first COVID-19 lockdown and propose some strategies that could be effective in fighting it during any future pandemic events. Materials and Methods: The study was conducted on patients with maxillofacial trauma who arrived at the Maxillofacial Unit of the Magna Graecia University of Catanzaro from 9 March to 3 May 2020, who were compared with those registered in the same period in 2019. Inclusion criteria were: patients of both sexes and admission diagnosed with maxillofacial trauma with or without bone fracture. Exclusion criteria were: patients less than 7 years of age, maxillofacial trauma that occurred outside the established period, and patients unconscious or with unclear clinical history. Patients were divided into two groups according to the mechanism of injury (MOI): “domestic” and “non-domestic” trauma. Both descriptive and regressive statistical analysis was conducted using a Student’s *t*-test with a significance level set at *p* < 0.05. Results: The total number of maxillofacial fractures in 2020 was similar to 2019 (31 pcs in 2020 vs. 38 pcs in 2019). Before the lockdown, most of the trauma occurred in non-domestic settings (25% in 2020 vs. 76.67% in 2019), especially in road accidents (4.17% in 2020 vs. 20% in 2019). During the lockdown, most of the trauma occurred in a domestic setting (75% in 2020 vs. 23.33% in 2019), especially interpersonal violence (31.58% in 2020 vs. 14.28% in 2019). There were 7 cases of interpersonal violence recorded in 2020 (1 male and 6 female), compared to only one case (female) recorded in 2019, with a statistically significant *p*-Value (0.0475). Conclusions: The first COVID-19 lockdown has provided the opportunity to study the aetiology of domestic trauma due to interpersonal violence attributable to economic and social problems, all of which were aggravated by the impediment to requesting help due to the difficulty of contacting the services and the general slowdown in the ways out of violence. The analysis conducted and compared with data in the literature suggests the adoption of a proactive (and non-reactive) approach to combat domestic violence during pandemic events.

## 1. Introduction

Since the World Health Organization (WHO) declared COVID-19 a pandemic, people’s lives have drastically changed due to the containment measures applied to stem the infections. All hospitals, including maxillofacial surgery departments, were involved in an immediate change in planning and working methods, with the suspension of scheduled activities [1,2]. In this way, the management of all trauma emergencies and not deferable disease was assured.

These measures were essential to defeat the virus and protect health systems, but were equally responsible for negative and unwanted consequences [3]. The isolation of people, the prohibition of crowding and the forced stay in one’s home (except for going out for essential needs) led to an increase in domestic trauma compared to non-domestic ones. Non-domestic traumas, especially from road accidents, represent the most frequent cause of hospitalizations in maxillofacial wards all over the world [4]. Many studies have examined how the lockdown has worked on mental health and its relationship to anxiety and depression [5,6,7,8,9]. Adams-Prassl et al. reported the adverse effects of quarantine on the mental health of the US population [10]; other researchers have found that lockdown affects sleep quality and that higher levels of anxiety can be explained by time spent reading and discussing COVID-19 news. Isolation, job loss (especially in families in already precarious economic situations), and the increase in alcohol abuse are all factors that have led to depression, dissatisfaction and anger [11]. All this has therefore worsened the conditions of women and children (the most affected subjects) who already lived in an unhealthy home environment. This would explain the increase in domestic trauma due to interpersonal violence during the first lockdown. The COVID-19 lockdown provided the opportunity to: (a) study the epidemiological effects of reversal of trauma; (b) demonstrate that the increase in domestic accidents during COVID-19 is an indirect driver of the economic and social crisis; (c) above all, to study what strategies can be put in place to combat the negative effects of any future pandemic events on domestic violence. Before the COVID-19 pandemic, victims of domestic violence had access to support from family and relatives, sheltered homes, and even legal remedies such as protection and restraining orders. Unfortunately, all of this was no longer readily available with the lockdown. Strengthening first response systems, ensuring that domestic violence is integrated into health response systems, expanding and strengthening social safety nets, offering shelter as temporary housing, encouraging temporary networks of social support, and integrating domestic violence could be great ideas to be taken [12]. Another strategy is certainly to improve the reporting system: the literature shows how the victim of domestic violence denounces only in case of removal from the perpetrator [13]. The pandemic interfered with this, as the victims were in close contact with their attackers. Scholars also argue that it is essential to train health personnel who already work in this field to better deal with these emergencies. This can make a significant difference in achieving higher reporting rates by encouraging community members to report cases of domestic violence in their neighbourhoods and by developing digital monitoring programs. The purpose of this retrospective study is to identify, through the experience reported by the authors, the potential reasons for the increase in maxillofacial trauma from domestic violence in the first COVID-19 lockdown compared to previous years and compare them with the data present in the literature and to propose some strategies that could be effective in the fight against domestic violence during any future pandemic events.

## 2. Materials and Methods

This is a retrospective study involving patients that occurred to our Maxillofacial Surgery Unit of the “Magna Graecia” University of Catanzaro in a period ranging from 9 March 2020 to 3 May 2020. Data collected were analyzed and compared to those belonging to the same period in the previous year (2019).

### 2.1. Inclusion and Exclusion Criteria

The study inclusion criteria included patients of both sexes, over seven years of age, and admission with a diagnosis of maxillofacial trauma with or without bone fracture.

The exclusion criteria were: patients younger than 7 years because of the absence of a pediatric intensive care unit in our hospital; maxillofacial trauma that occurred outside the established period; and unconscious patients or patients with unclear clinical history.

### 2.2. Search Strategy

The primary objective was to verify the incidence of domestic maxillofacial traumas compared to non-domestic ones in the two periods examined. A comparison was also made based on the injury mechanism.

The secondary objective was to verify whether domestic violence had a different incidence as a mechanism of injury in maxillofacial trauma in the two periods examined and to identify the causes.

According to the mechanism of injury (MOI), patients with bone fractures were divided into two groups: “domestic trauma” (violence, scuffles, accidental falls, and attempted suicide) and “non-domestic trauma” (road accidents with or without motor vehicle involvement). Bone fractures were all classified according to the AO-CMF Criteria [14]. All patients arrived at our surgical unit from the emergency departments of other local Hospitals. They all arrived stabilized, screened by nasopharyngeal swab for SARS-CoV-2, and subjected to radiological exams and urgent specialist visits. After clinical and radiological consultation, hospitalisation was made in case of surgical indication. Before our admission, a second triage (questioning about symptoms and contacts with COVID-19-positive people) and a second nasopharyngeal swab were performed (the patient was placed in isolation pending the swab result).

Results were compared to data deriving from the analysis of traumas registered in 2019, in the same period we took into consideration for 2020, and with the same inclusion and exclusion criteria. It should be specified that the data trend for 2019 was entirely comparable to that of the previous two years (2017 and 2018).

### 2.3. Trial Procedures

This study followed the Helsinki Declaration on Medical Protocol and Ethics. The study was approved by the Ethics Committee of Magna Graecia University of Catanzaro (reference number 146 of 21 May 2020). 

### 2.4. Statistical Analysis

Both descriptive and regressive statistical analyses were performed on the recorded data. Descriptive statistical analysis was performed using central tendency indices (such as mean and range) and absolute and relative frequencies for categorical data. Regressive statistical analysis was performed using Student’s *t*-test calculated using the GraphPad program (GraphPad Company, San Diego, CA, USA).

## 3. Results

Thirty-one patients were evaluated as trauma to our maxillofacial surgical unit during the COVID-19 lockdown (from 9 March to 3 May 2020). According to the mechanism of injury (MOI), patients with bone fractures were divided into two groups: (1) domestic trauma (interpersonal violence, accidental falls, and attempted suicide); (2) non-domestic trauma (interpersonal violence, scuffles, accidental falls, road accidents with or without motor vehicle involvement). Data relating to MOI of both groups are schematized in Table 1. Instead, data relating to the site of fractures, based on AO-CMF classification, are schematized in Table 2.

Two cases (6.45%) did not have maxillofacial bone fractures; all of these suffered in the domestic setting (accidental falls).

Five cases (16.13%) suffered from compound fractures that did not require surgical treatment. Of all these, 3 cases (60%—1 male and 2 females) suffered domestic trauma, and 2 cases (40%—1 male and 1 female) suffered non-domestic trauma. Three patients (60%) presented with compound nose fractures, and two patients (40%) presented with compound OMZ complex fractures.

A total of 24 patients (77.42%) underwent surgical procedures under general anaesthesia for maxillofacial bone fractures during the COVID-19 lockdown, 11 males (45.84%) and 13 females (54.16%), with a mean age of 63.36 (range 9–87).

In the timeframe analyzed, a total of 19 patients (61.29%) presented with a maxillofacial bone fracture that occurred in the domestic setting; 16 of these patients needed surgical treatment. Furthermore, 10 patients (32.26%) presented with a maxillofacial bone fracture that occurred outside; 8 of these patients needed surgical treatment.

Of all patients who did not need surgery, 1 male (20%) suffered domestic trauma (accidental fall), and 1 male (20%) suffered non-domestic trauma (road trauma); furthermore, 2 females (40%) suffered domestic trauma (1 interpersonal violence and 1 accidental fall), and 1 female (20%) suffered non-domestic trauma (road trauma).

Of all patients undergoing surgery, 5 males (20.83%) suffered domestic trauma (1 interpersonal violence, 1 attempted suicide, and 3 accidental falls), and 6 males (25%) suffered non-domestic trauma (2 accidental falls, 3 road trauma, and 1 road trauma not related to motor vehicles); furthermore, 11 females (45.83%) suffered domestic trauma (5 interpersonal violence and 6 accidental falls), and 2 females (8.34%) suffered non-domestic trauma (1 accidental fall and road trauma).

Regarding trauma due to interpersonal violence, we registered a total of 7 cases in the domestic setting, precisely 1 male (14.28%) and 6 females (85.72%), in 2020. One patient attempted suicide in the domestic setting, reporting a pan-facial fracture. Most cases of traumas were related to accidental falls (16 cases—51.61%), both in domestic (11 cases: 4 males and 7 females) and non-domestic (5 cases: 4 males and 1 female) settings. According to the MOI, we reported 7 cases (22.58%) of road trauma with or without motors; these traumas were only recorded as expected in the non-domestic setting. Precisely, 5 patients reported surgically treated fractures (3 males and 1 female presented with maxillofacial fractures after a vehicle-related traffic injury; 1 male presented with a maxillofacial fracture after a non-vehicle-related traffic injury while jogging), and 2 patients reported compound fractures (1 male and 1 female).

According to Table 2, the most frequent fracture involves the orbit, both individually and in the orbit-maxilla-zygomatic complex (OZM), in both domestic and non-domestic settings. Furthermore, OZM complex and orbit fractures are more frequent in the domestic setting than in non-domestic ones: 11 cases (3 males and 8 females) in domestic settings compared to 5 cases (3 males and 2 females) in non-domestic settings. The second in frequency is jaw fracture, especially in females and in the domestic setting.

The data for 2020 were compared with the data for 2019 in the same period. The data of surgically treated patients in this period are comparable to the previous two years, both in terms of the total number of surgery and in terms of the proportions of domestic and non-domestic trauma. In 2019, thirty-eight patients were evaluated for maxillofacial traumas: thirty patients (78.95%) underwent surgical procedures under general anaesthesia for maxillofacial bone fractures; eight patients (21.05%), instead, had compound bone fractures. Of all surgically treated patients, only 7 (23.33%) suffered domestic trauma, precisely, 4 males (all accidental falls in elderly patients) and 3 females (1 interpersonal violence and 2 accidental falls). Only one case was related to interpersonal violence and was recorded in a woman.

Of all patients undergoing surgery for non-domestic trauma, 5 patients (16.66%—4 males and 1 female) suffered work accidents, 3 patients (10%—3 males) suffered sports accidents, 6 patients (20%—3 males and 3 females) suffered vehicle-related traffic injury, and 2 patients (6.66%—1 male and 1 female) suffered non-vehicle-related traffic injury.

The total number of maxillofacial fractures treated surgically in 2020 is comparable to that of the previous year. Before the COVID-19 lockdown, the most common mechanism of maxillofacial trauma was related to non-domestic settings (25% in 2020 vs. 76.67% in 2019), particularly road accidents (4.17% in 2020 vs. 20% in 2019). On the other hand, during the COVID-19 lockdown, the most common mechanism of maxillofacial trauma was related to domestic settings (75% in 2020 vs. 23.33% in 2019), in particular interpersonal violence (31.58% in 2020 vs. 14.28% in 2019). Data relating to domestic trauma in 2019 and 2020 are schematized in Table 3.

Analysis of Student’s *t*-test was applied for the two years 2019–2020, showing a statistically significant increase in traumas in the domestic setting for 2020 (*p*-value = 0.0475), especially in domestic violence.

## 4. Discussion

The lockdown imposed by the COVID-19 pandemic has led to the isolation of people and a ban on leaving your house for unnecessary reasons, resulting in a reduction in the number of non-domestic trauma [4,15,16]. The statistical analysis conducted by the authors shows a much lower percentage of domestic trauma in 2019 than in 2020; these were mainly due to accidental falls of elderly patients (76.67% in 2019 vs. 25% in 2020). This is also reported in other studies that attest a decrease in the rate of road accidents and sports-related injuries (4.17% in 2020 vs. 20% in 2019) due to the confinement and isolation measures adopted by governments [1,17,18,19,20]. The most important issue is the increase in domestic trauma from interpersonal violence, especially against women and children, recorded in 2020 compared to the previous year.

Previous outbreaks of infectious diseases (such as SARS and Ebola) had already shown their psychological impact, characterized by widespread fear, anxiety, and other psychological affections [21]. Our experience attests an increase in cases of domestic trauma (75% in 2020 vs. 23.33% in 2019) during the lockdown phase. It confirms that this increase is caused by social and economic changes, which, as reported in the literature, induces depression and the development of interpersonal violence at a much higher percentage than the previous year taken as a reference.

Based on this increase in maxillofacial trauma in the domestic settings, we wanted to focus on those related to domestic violence (31.58% in 2020 vs. 14.28% in 2019). There were 7 cases of interpersonal violence recorded in 2020 (1 male, 14.28%, and 6 female, 85.72%), compared to only one case (female) recorded in 2019, with a statistically significant p-value (0.0475).

By definition, domestic violence refers to a series of violations that occur within the home setting; the mainly affected subjects are women and children. It is a broad term that encompasses intimate partner violence, a form of abuse perpetrated by a current or former partner [22,23,24,25,26]. The increase in maxillofacial trauma due to domestic violence reported in this study, even if it is the expression of a local trend, follows what has been observed at a global level [27,28]. An increase in domestic violence episodes has recently been shown, with a relative increase in calls to assistance services within a week of adopting strict social distancing and blocking measures: around 40-50% in Brazil, 20-30% in Spain, and 25% in the UK. This global trend was also encountered in the USA: about 22% in Portland and 10% in New York City [28,29].

Therefore, an important question arises: “what strategies can be put in place to prevent the negative effects of COVID-19 on domestic violence?” It is essential to reassure women that the anti-violence network is present, active and able to support them even in times of pandemics. They should be able to continue to receive advice, support and protection. It is also essential to raise awareness among the population on the importance of contacting the police if they witness situations of violence. During the compulsory confinement phases induced by the COVID-19 pandemic, the government should have launched an information campaign to combat gender-based violence and domestic violence, also by sharing resources and information for victims, with the possibility for the latter to go out to look for help without facing sanctions.

Of course, the retrospective study reported and analysed by the authors is not based on a large case study, and this may represent a limitation in the analysis presented and in the suggested strategy. Further investigations will certainly be needed in the future and comparisons with other studies in the literature.

Certainly, we cannot put what we have experienced behind us and we need to consider the effects that the pandemic had on society. These effects are most likely not going to be short-lived, especially for those people who have experienced domestic violence and abandonment of society [20,30]. Therefore, it is essential to learn from the pandemic, and in particular from the lockdown, as an unexpected scientific advance promoted by the work of scholars of every discipline (medical, biomedical, humanities, etc.) who have focused on research to generate new ideas and new resources. Now it becomes essential to transform this experience into a sustainable development challenge, looking to the future with the awareness that any future pandemic event will not find us unprepared.

## 5. Conclusions

Global restrictive measures have been implemented in order to stem the spread of COVID-19 and flatten the curve of the pandemic. However, this lockdown has had numerous consequences on people’s lives: major economic and social changes, changes in education and sociability, general insecurity, global economic depression, an increase in eating and mental health disorders and, not least, increased trauma in the home. It has generated a paradox regarding the security of staying at home. Our work highlights how domestic traumas have increased, especially those due to domestic violence (it mainly affects women and children), as it emerged from other studies reported in the literature. Another negative consequence of these restrictive measures is the reduction in the number of women seeking assistance. While indispensable, these measures have reduced women’s chances of seeking help from anti-violence centres and/or emergency services. There is, therefore, a clear need, both at a national and local level, to act both in terms of support and protection of victims of domestic violence. It is essential to implement local responses and telemedicine consultations to facilitate the recognition of domestic violence and allow a faster response.

## Figures and Tables

**Table 1:** Subdivision of patients according to the mechanism of injury and by domestic and non-domestic settings.

Maxillofacial Traumas from 9 March to 3 May 2020 Total Patients: 31								
MOI Mechanism of Injury	Domestic Trauma				Non-Domestic Traumas			
	Male		Female		Male		Female	
	N°	%	N°	%	N°	%	N°	%
**Surgically treated patients: 24**								
Interpersonal violence	1	4.17%	5	20.82%	-	-	-	-
Attempted suicide	1	4.17%	-	-	-	-	-	-
Accidental falls	3	12.5%	6	25%	2	8.33%	1	4.17%
Road trauma related to motor	-	-	-	-	3	12.5%	1	4.17%
Road trauma not related to motor	-	-	-	-	1	4.17%	-	-
**Patients who did not require surgery: 5**								
Interpersonal violence	-	-	1	20%	-	-	-	-
Attempted suicide	-	-	-	-	-	-	-	-
Accidental falls	1	20%	1	20%	-	-	-	-
Road trauma related to motor	-	-	-	-	1	20%	1	20%
Road trauma not related to motor	-	-	-	-	-	-	-	-
**Patients who did not have fractures: 2**								
Interpersonal violence	-	-	-	-	-	-	-	-
Attempted suicide	-	-	-	-	-	-	-	-
Accidental falls	2	100%	-	-	-	-	-	-
Road trauma related to motor	-	-	-	-	-	-	-	-
Road trauma not related to motor	-	-	-	-	-	-	-	-

**Table 2:** Subdivision of patients according to the type of fracture based on AO-CMF classification.

Site of Fracture								
	Domestic Trauma: 19				Non-Domestic Trauma: 12			
	Male		Female		Male		Female	
	N°	%	N°	%	N°	%	N°	%
**Surgically treated patients: 24**								
Orbito-maxillo-zygomatic complex (OMZ)	2	8.33%	4	16.67%	1	4.17%	1	4.17%
Orbit	1	4.17%	4	16.67%	2	8.33%	1	4.17%
Naso-orbito-ethmoidal complex (NOE)	1	4.17%	1	4.17%	1	4.17%	-	-
Nose	-	-	-	-	-	-	-	-
Jaw	1	4.17%	2	8.33%	1	4.17%	-	-
Pan facial	-	-	-	-	1	4.17%	-	-
**Patients who did not require surgery: 5**								
Orbito-maxillo-zygomatic complex (OMZ)	-	-	2	40%	1	20%	-	-
Orbit	-	-	-	-	-	-	1	20%
Naso-orbito-ethmoidal complex (NOE)	-	-	-	-	-	-	-	-
Nose	1	20%	-	-	-	-	-	-
Jaw	-	-	-	-	-	-	-	-
Pan facial	-	-	-	-	-	-	-	-
**Patients who did not have fractures: 2**								
Orbito-maxillo-zygomatic complex (OMZ)	-	-	-	-	-	-	-	-
Orbit	-	-	-	-	-	-	-	-
Naso-orbito-ethmoidal complex (NOE)	-	-	-	-	-	-	-	-
Nose	2	100%	-	-	-	-	-	-
Jaw	-	-	-	-	-	-	-	-
Pan facial	-	-	-	-	-	-	-	-

**Table 3:** Comparison of traumas that occurred in domestic settings in the timeframe 9 March to 3 May of 2020 with 2019.

Maxillofacial Traumas from 9 March to 3 May 2019 and 2020								
MOI Mechanism of Injury	Domestic Traumas of 2019				Domestic Traumas of 2020			
	Male		Female		Male		Female	
	N°	%	N°	%	N°	%	N°	%
Interpersonal violence	-	-	1	14.28%	1	4.17%	5	20.82%
Attempted suicide	-	-	-	-	1	4.17%	-	-
Accidental falls	4	57.14%	2	28.58%	3	12.5%	6	25%

## Data Availability

The data presented in this study are available on request from the corresponding author.
